# Validation of the Updated Alternate Fistula Risk Score for Prediction of Postoperative Pancreatic Fistula After Pancreatoduodenectomy

**DOI:** 10.1002/jgh3.70053

**Published:** 2024-11-26

**Authors:** Yugal Limbu, Bidur Prasad Acharya, Sneha Raut, Sujan Regmee, Roshan Ghimire, Dhiresh Kumar Maharjan, Prabin Bikram Thapa

**Affiliations:** ^1^ Department of Gastrointestinal and General Surgery Kathmandu Medical College Teaching Hospital Kathmandu Nepal; ^2^ Kathmandu Medical College Teaching Hospital Kathmandu Nepal

**Keywords:** original fistula score, pancreatic duct, pancreatic fistula, pancreatoduodenectomy, updated alternative fistula score

## Abstract

**Background and Aim:**

Postoperative pancreatic fistula (POPF) remains a significant challenge following pancreatoduodenectomy (PD), contributing to morbidity and mortality. Various risk assessment models have been established to predict the likelihood of POPF. An updated alternate fistula risk score (ua‐FRS) has been recently refined and validated within European cohorts. However, the validation of this score in South Asian cohorts remains relatively unexplored. This study aims to validate the applicability of ua‐FRS for the prediction of POPF in patients undergoing PD in the South Asian population, particularly Nepal.

**Methods:**

This cross‐sectional, observational study was conducted by a single team across three tertiary care centers in Kathmandu, Nepal from July 2021 to October 2023. A total of 98 patients were studied in terms of their sex, body mass index (BMI), diameter of the main pancreatic duct (MPD), pancreatic consistency, pathological site, and estimated blood loss. The accuracy of ua‐FRS for the prediction of postoperative pancreatic fistula after pancreatoduodenectomy was evaluated using the receiver operative characteristics curve.

**Results:**

Univariate analysis revealed that sex, pancreatic gland texture, the diameter of the main pancreatic duct, the site of pathology, and BMI were statistically significant factors. However, in the multivariate analysis, only BMI and the diameter of the MPD retained their statistical significance, with *p*‐values less than 0.005. The ua‐FRS demonstrated high sensitivity and specificity in predicting postoperative pancreatic fistula, as evidenced by an area under the curve (AUC) of 0.802.

**Conclusion:**

The ua‐FRS has validation in the context of the South Asian population to predict POPF following PD, offering a reliable tool to guide perioperative management.

## 1 Introduction

Post‐operative pancreatic fistula (POPF) significantly causes perioperative morbidity and mortality after pancreatoduodenectomy (PD) [1, 2]. Several risk assessment models have been developed to predict the likelihood of POPF [3, 4]. Among these, the original fistula risk score (o‐FRS) proposed by Ryu et al. [5] stands out as the most widely utilized and validated model for predicting this complication.

The o‐FRS is based on pancreatic texture, duct diameter, blood loss, and pathology and was designed and validated in the US population [6]. The alternative FRS (a‐FRS) model was based on pancreatic texture, duct diameter, and body mass index (BMI), designed in a European population and subsequently validated in Europe and the US [4]. The a‐FRS did not include blood loss as this variable was thought to be less reliable [4]. Recently, an updated version of a‐FRS (ua‐FRS) was described which added the male sex as a variable and was validated in a European population [7]. These scoring systems have been used to augment perioperative management such as the decision of drain placement, timings of drain removal, and prophylactic use of somatostatin analogues [3, 4, 7, 8, 9].

The o‐FRS, known for its reliability in Western populations, has demonstrated varying results when applied to Eastern cohorts, underscoring the need for region‐specific validation studies [10, 11, 12, 13]. Similarly, while ua‐FRS has been recently validated in the European population, its effectiveness in the South Asian context remains unexplored [7]. This study aims to validate the applicability of ua‐FRS in predicting the incidence of clinically relevant postoperative pancreatic fistula (CR‐POPF) in patients undergoing PD in the South Asian population, while also exploring cohort‐specific factors associated with increased risk.

## 2 Method

### 2.1 Study Design

This is a cross‐sectional, observational study involving 98 patients who underwent PD, conducted by a single team across three hospitals in Kathmandu, inclusive of Kathmandu Medical College Teaching Hospital, Grande International Hospital, and Nepal Cancer Hospital and Research Center, from July 2021 to October 2023. Ethical approval was obtained from the Kathmandu Medical College Institutional Review Committee (Ref: 10062021204) for all participating centers.

### 2.2 Sample Size Determination

**FIGURE 1:**
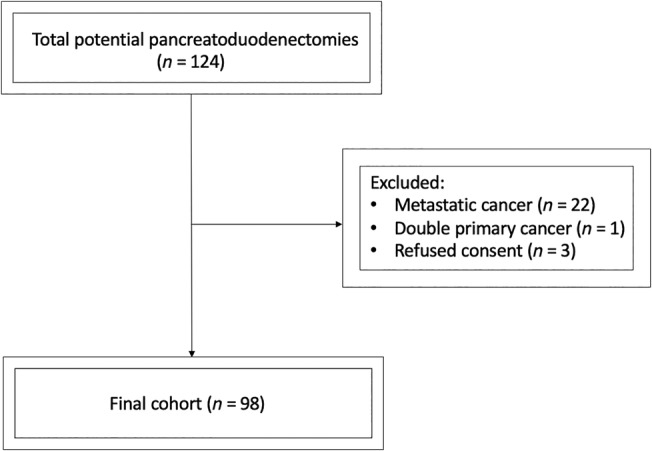
Flowchart illustrating patient enrollment and inclusion process.

The determination of the sample size for this study was based on the prevalence rate of CR‐POPF as reported in the study conducted by Smits et al. [14] The formula utilized for sample size calculation was *n* = (*Z* ^2^ * *p* * *q*)/*e* ^2^, where, *Z* represents the standard normal deviation for a desired confidence level (in this case, 1.96 for a 95% confidence interval), *p* denotes the prevalence of CR‐POPF obtained from the referenced study (0.12) [14], *q* is the complementary probability of *p* (1 − *p*, which equals 0.88), and *e* signifies the margin of error (10%). Substituting the respective values into the formula, the calculated sample size was determined to be 41. However, we included all 98 patients meeting the inclusion criteria by using the purposive sampling method (Figure 1).

### 2.3 Inclusion Criteria

Patients over the age of 18 years who underwent PD.

### 2.4 Exclusion Criteria

Patients with metastatic cancer, dual primary cancer and those who refused consent.

### 2.5 Data Collection

Comprehensive data collection on the pancreatoduodenectomies included specific peri‐operative details and postoperative outcomes. Informed consent was obtained from all patients. The duct‐to‐mucosa technique utilizing the Blumgart technique was used for pancreatico‐enteric anastomosis during reconstruction. Polydioxanone 5–0 sutures were used for duct‐to‐mucosa anastomosis and polypropylene 3–0 sutures were used for trans‐pancreatic sutures. Octreotide was prophylactically administered, and the use of a transanastomotic stent was determined based on intraoperative findings and the surgeon's discretion.

The preoperative assessment encompassed patient demographics including BMI, symptomatology, laboratory results, imaging findings, and pre‐operative treatments. Intraoperatively, factors such as estimated volume of blood loss, surgery duration, quantity of fluid administration, necessity for and number of blood transfusions, and main pancreatic duct (MPD) diameter were meticulously documented. MPD diameter was measured by inserting feeding tubes of varying sizes that snugly fit the MPD, and subsequently, the French gauge (Fr) size of the tube was converted to the corresponding diameter. During the surgery, the experienced surgeon subjectively assessed the texture of the pancreatic gland based on tactile sensations and visual appearance. The surgeon recorded observations regarding the gland's texture, categorizing it as soft or firm. Postoperatively, clinical outcomes including incidence, type, and severity of complications including POPF, the duration of intensive care unit (ICU) stay, total duration of hospital stay, and any hospital readmissions or reoperations within 90 days were recorded.

Mortality was defined as death during the initial hospitalization or within 30 days of hospital discharge, or death due to any surgical complication at any point in time. POPF probability in each case was calculated using the ua‐FRS from the online calculator obtained from www.pancreascalculator.com. A calibration plot was used to represent the predicted risk against the observed risk for POPF based on ua‐FRS models. Calibration assesses the ability to correctly quantify the observed absolute risk.

### 2.6 Variables

The dependent variable was determined by the ua‐FRS score, which served as a predictive measure for POPF following PD. On the other hand, the independent variables encompassed sex, MPD diameter, pancreatic consistency, BMI, pathological site, and estimated blood loss. These independent variables were considered for their potential influence on the outcome of interest, allowing us to explore the multifaceted aspects contributing to POPF risk after the surgical procedure.

### 2.7 Statistical Analysis

The data entry was done in Microsoft Excel and Statistical Package for Social Sciences (SPSS) version 26 (IBM Inc. Armonk, NY, USA) [15] was used for statistical analysis. Categorical data were analyzed using the chi‐square test. The diagnostic values of the o‐FRS, a‐FRS, and ua‐FRS were analyzed by calculating the area under the receiver operating characteristics (AUROC) curves. The model's performance was assessed through ROC and calibration plots. Continuous variables were expressed as median (inter‐quartile range). For regression assessment, univariate and multivariate logistics regression analysis was used. A *p*‐value of < 0.05 was considered significant.

## 3 Results

### 3.1 Demography

During the study period, a total of 98 pancreatoduodenectomies were performed by a single team in various centers for periampullary pathologies. The median age was 60 years (interquartile range [IQR] 54.75–67.50 years), and the median BMI was 22.30 kg/m^2^ (IQR 20.80–23.20 kg/m^2^). The male‐to‐female ratio was approximately 1.72–1. Diabetes mellitus was present in 25 (25.51%) patients, hypertension in 38 (38.77%), and Chronic Obstructive Pulmonary Disease in 9 (9.18%) patients.

### 3.2 Intraoperative Data

Most patients had an MPD size of 4 mm, accounting for 56 (57.14%) of the total patients. Among the 98 patients, 54 (55.10%) had a pancreas with a hard‐to‐firm consistency, and 64 (65.31%) experienced intraoperative estimated blood loss of less than 400 mL.

### 3.3 Postoperative Data

Postoperative pathology confirmed that pancreatic ductal adenocarcinoma (PDAC) was the most common pathology, accounting for 46 (46.94%) patients. A majority of 75 (76.53%) patients successfully avoided the development of a POPF, while 23 (23.47%) encountered this complication, including incidents of biochemical leakage (Grade A POPF). One (1.02%) patient underwent re‐exploration due to a Gastroduodenal artery pseudoaneurysm after unsuccessful endovascular coiling, which later resulted in mortality. Other complications included surgical site infections (SSI) in five (5.10%) patients and delayed gastric emptying in 18 (18.36%) patients. A mortality rate of 4.08% was recorded. Detailed characteristics and risk factors for POPF are provided in Table 1.

**TABLE 1:** Baseline characteristics, fistula risk factors, and additional treatments for postoperative pancreatic fistula.

Variables	Number (*N*)/percentage (%)	Variables	Number (*N*)/percentage (%)
Age	58.1 ± 15.20	Body mass index	21.97 ± 2.15
Sex		Gland texture	
Male	62 (63.27%)	Firm	54 (55.10%)
Female	36 (36.73%)	Soft	44 (44.90%)
Pancreatic duct size		Intraoperative blood loss	
> 5 mm	56 (57.14%)	< 400 mL	64 (65.31%)
4	15 (15.31%)	401–700 mL	25 (25.51%)
3	17 (17.35%)	701–999 mL	7 (7.14%)
2	6 (6.12%)	> 1 L	2 (2.04%)
< 1	4 (4.08%)	Pancreatic fistula	
Pathology		No fistula	75 (76.53%)
Pancreatic ductal adenocarcinoma	46 (46.94%)	Biochemical leak	14 (14.29%)
Others	52 (53.06%)	Clinically relevant postoperative pancreatic fistula	
Ampullary carcinoma	26	International Study Group for Pancreatic Fistula Grade B	6 (6.12%)
Distal cholangiocarcinoma	12	International Study Group for Pancreatic Fistula Grade C	3 (3.06%)
Chronic pancreatitis with head mass	6	Additional treatment	
Intraductal papillary mucinous neoplasm	4	Total parenteral nutrition	2 (2.04%)
Duodenal adenocarcinoma	2	Percutaneous drainage	4 (4.08%)
Pancreatic neuroendocrine tumors	2	Antibiotics use	11 (11.22%)
		Re‐exploration	3 (3.06%)
		Aneurysmal clipping	2 (2.04%)

### 3.4 Statistical Analysis and Diagnostic Value Assessment

In the univariate analysis, all factors, except for estimated blood loss, exhibited statistical significance with *p*‐values less than 0.05. This indicates a significant association between BMI (≥ 25 kg/m^2^), MPD (per mm increase), pancreatic gland texture (firm), pathology (not PDAC), and male gender with the incidence of clinically relevant POPF. In the multivariate analysis, BMI and MPD diameter were statistically significant, with *p*‐values of 0.019 and 0.006, respectively. Detailed odds ratios and 95% confidence intervals for both univariate and multivariate analyses are provided in Table 2.

**TABLE 2:** Univariate and multivariate logistic regression analysis of fistula risk score factors associated with postoperative pancreatic fistula following pancreatoduodenectomy.

Variables	Univariate			Multivariate		
	Odds ratio	95% Confidence interval	*p* ^a^	Odds ratio	95% Confidence interval	*p* ^a^
Male gender	3.54	1.10–11.4	0.035	2.84	0.72–11.22	0.14
Body mass index (≥ 25 kg/m^2^)	1.63	1.21–2.19	0.001	1.52	1.07–2.15	0.019
Pancreatic gland texture (firm or hard)	0.34	0.12–0.97	0.043	0.58	0.15–2.23	0.43
Main pancreatic duct size (per mm increase)	0.40	0.26–0.61	< 0.0001	0.52	0.32–0.82	0.006
Pathology (not pancreatic ductal adenocarcinoma)	3.24	1.15–9.12	0.026	1.86	0.45–7.43	0.38
Estimated blood loss	1.00	1–1.004	0.192	1.001	1–1.005	

^a^ *p*‐value derived from Pearson's Chi‐squared test.

**FIGURE 2:**
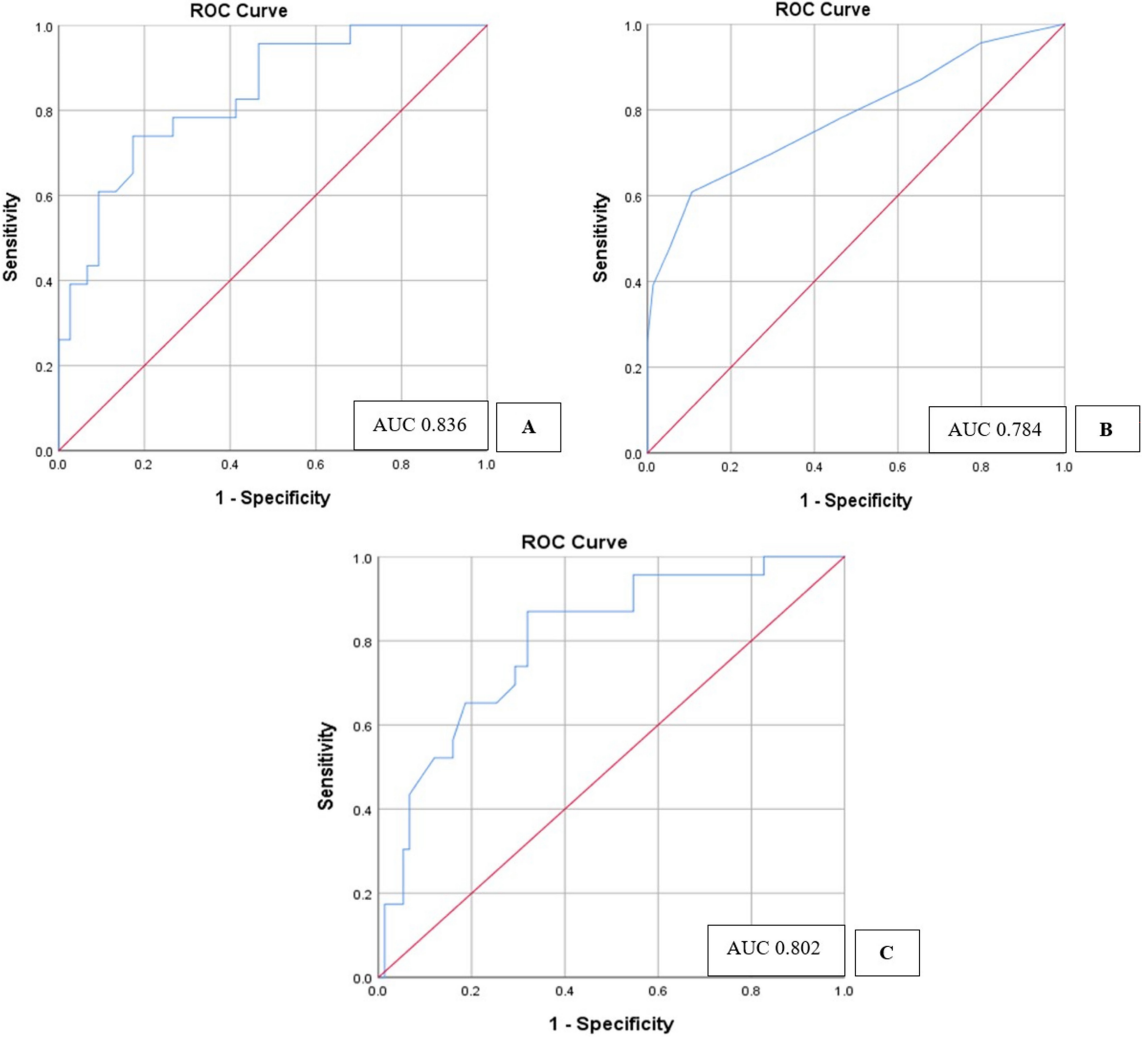
Receiver operating characteristics (ROC) curves and the area under the curve (AUC) for (A) original fistula risk score, (B) alternative fistula risk score, and (C) updated alternative fistula risk score.

The area under the curve (AUC) was similar in o‐FRS, a‐FRS, and ua‐FRS with AUC of 0.836, 0.784, and 0.802, respectively which shows the diagnostic value of each fistula risk scores that are similar in predicting the POPF (Figure 2). Notably, the a‐FRS demonstrated the highest specificity (82.7%), while the ua‐FRS showed the highest sensitivity (87%). Additionally, the ua‐FRS had the highest negative predictive value (94.4%), reflecting its ability to accurately predict the absence of POPF (Table 3).

**TABLE 3:** Comparison of diagnostic performance between the alternative fistula score (a‐FRS), original fistula score (o‐FRS), and updated alternative fistula score (ua‐FRS).

Score	Sensitivity (%)	Specificity (%)	PPV^a^ (%)	NPV^b^ (%)
Original fistula risk score (o‐FRS)	60.9	89.3	63.6	88.2
Alternate fistula risk score (a‐FRS)	73.9	82.7	56.7	91.2
Updated alternate fistula risk score (ua‐FRS)	87	68	45.5	94.4

^a^ Positive predictive value.

^b^ Negative predictive value.

## 4 Discussion

Our study validated the ua‐FRS, demonstrating its statistical significance across all individual components within the scoring system. The three fistula scores (o‐FRS, a‐FRS, and ua‐FRS) exhibit similar diagnostic performance in predicting POPF, with AUC values of 0.836, 0.784, and 0.802, respectively. A previous study on eastern cohorts, reported lower AUC values for the fistula risk scores [13]. Another prior study reported that the predictive performance of the a‐FRS was similar to the original FRS, both under the 2005 definition (AUC 0.78 vs. 0.75, *p* = 0.03) and the 2016 definition (AUC 0.72 vs. 0.70, *p* = 0.05) [4]. Given the similar diagnostic performance of all scores in our study, no significant superiority exists among them in this specific population. Therefore, surgeons may consider any of the three scores for predicting POPF in the South Asian population. The ua‐FRS excelled in sensitivity and negative predictive value, making it useful for ruling out POPF, while the o‐FRS had the highest specificity and positive predictive value, indicating its strength in confirming cases of POPF. The a‐FRS, on the other hand, offered a balanced profile with a strong combination of sensitivity and specificity. Multivariate analysis identified BMI and MPD diameter as significant predictors of POPF, emphasizing their clinical relevance (*p*‐value < 0.005).

The o‐FRS and a‐FRS have been validated in numerous studies, establishing themselves as precise tools for predicting POPF following PD [12, 16]. Initially, these validations did not yield statistically significant results due to single‐centered enrollments and insufficient sample sizes. However, these issues have been addressed over time, resulting in more robust validation. While earlier studies predominantly examined European and American cohorts, our study stands out as potentially the first to explore the validity of this predictive score for POPF in the South Asian setting [17].

In our study, soft pancreatic texture emerged as a risk factor for CR‐POPF in univariate analysis but lost prognostic independence in multivariate analysis, potentially due to the relatively small sample size and potential subjective evaluation biases. MPD diameter emerged as a significant determinant of POPF in both analyses, consistent with previous findings indicating that MPD diameter measuring over 3 mm has an inverse relationship with POPF incidence, thereby supporting the conclusion drawn from our data [17, 18]. Furthermore, a meta‐analysis showed that pancreatic duct diameter < 3 mm may increase the risk of POPF by 1.87 times [19]. Similarly, BMI has emerged as an independent risk factor for POPF development, likely due to its impact on visceral fat distribution, which plays a pivotal role in POPF occurrence [17, 18]. A sensitivity analysis elucidated that obesity class 1/2 and class 3 severe obesity are both associated with increased susceptibility to POPF [20].

Research has consistently demonstrated that the proficiency of the surgeon, operation within specialized hepatopancreatobiliary centers, and the execution of procedures in high‐volume surgical settings are all fundamental in impacting the rates of surgical complications [6, 7, 10]. A prior study implies that pancreatic pathology is a risk factor for pancreatic leakage [21]. While some studies suggest that the choice of pancreatico‐enteric anastomosis technique may impact the incidence of POPF, larger meta‐analyses have not corroborated these findings, indicating a lack of consensus on the technique's influence on surgical outcomes [18].

When evaluating the efficacy of different fistula risk scores, it is crucial to acknowledge that no scoring system is without limitations. This study's validity is subjected to a few limitations. First, the sample size of 98 patients, derived from three tertiary care centers in Kathmandu, Nepal, may not be representative of the entire South Asian population. As such, the generalizability of our findings to other regions or broader demographics may be limited. Consequently, while our results provide valuable insights into the predictors of POPF in the studied cohort, caution should be exercised in applying these findings universally. Future studies involving larger and more diverse populations are essential to validate our conclusions and enhance their applicability across different settings. Second, variability in perioperative care and data recording across different centers could lead to potential misclassification of both exposures and outcomes. However, the likelihood of this is mitigated given that a single surgical team conducted all procedures and the collection of data. Second, the o‐FRS study had adhered to the International Study Group for Pancreatic Surgery (ISGPS) 2005 definition for POPF in their original cohort, and it was not feasible to reclassify these cases according to the updated ISGPS 2016 criteria retrospectively which was utilized in our study. Finally, pancreatic texture assessment is inherently subjective and can vary between observers. Nonetheless, the impact of this bias is minimized in our study, as surgeons who have performed a high volume of pancreatic surgeries participated in the research, enhancing the uniformity and reliability of the assessment.

## 5 Conclusion

Our investigation successfully validates the updated a‐FRS within the South Asian population for the prediction of POPF following PD. This validation underlines the score's utility as a reliable tool in predicting POPF, offering clinicians a robust framework to individualize patients and potentially modify perioperative management strategies.

## Ethics Statement

This study was reviewed and approved by the Kathmandu Medical College Institutional Review Committee (Ref: 10062021204).

## Consent

All study participants, or their legal guardians, provided informed consent prior to study enrollment.

## Conflicts of Interest

The authors declare no conflicts of interest.

## Data Availability

Data are available upon reasonable request from the corresponding author.
